# Influence of Social Media Use on Behavioural Addiction and Social Isolation Among Healthcare Students: A Cross-Sectional Study

**DOI:** 10.7759/cureus.108771

**Published:** 2026-05-13

**Authors:** Anchal Gupta, Sandeep Arya, Pradeep Kumar, Chingakham Babita Devi, Anjali Gupta, Deblina Roy

**Affiliations:** 1 College of Nursing, King George's Medical University, Lucknow, IND; 2 College of Nursing, Uttar Pradesh University of Medical Sciences, Saifai, IND

**Keywords:** behavioral addiction, healthcare students, loneliness, social-isolation, social media addiction

## Abstract

Background

Problematic social media use has become an important public health concern worldwide. Its influence is even seen among healthcare students, who rely highly on digital platforms for academic, professional, and social engagement. Behavioural addiction is linked with excessive social media use and poor psychological well-being. The aim of this study is to assess the impact of social media-related behavioural addiction and loneliness and to identify the determinants of problematic social media use and loneliness among healthcare students.

Materials and methods

A cross-sectional study was conducted among healthcare students, with 200 participants. The Bergen Social Media Addiction Scale (BSMAS) and the UCLA Loneliness Scale (Version 3) were used for data collection. Mann-Whitney U test, Spearman’s correlation, and multiple linear regression were used to analyse the data.

Results

The mean Bergen Social Media Addiction and UCLA loneliness scores were 15.8 ± 4.71 and 43 ± 13.9, respectively. A significant positive correlation (rs = 0.325, p < 0.001) between problematic social media use and loneliness was found. Duration of daily social media use was the strongest predictor of problematic social media use (β = 0.31, p < 0.001), while family relationship detachment was a significant predictor (β = 0.21, p = 0.003) of loneliness. It was found that the duration of social media use is linked with addiction-like symptoms, and participants who have family detachment reported elevated loneliness.

Conclusion

The findings highlight the importance of targeted interventions such as digital literacy and the promotion of healthy and balanced social media use for overall mental health and well-being, which aligns with Sustainable Development Goal 3.

## Introduction

In 2025, worldwide social media users have increased to 5.24 billion, representing 63.9% of the global population. India has a population of 1.42 billion, with a rapid rise in active social media users [1]. Young adults, especially students, are surrounded by a plethora of distractions, which not only affect their academic performance but also increase the risk of behavioural addiction. Problematic social media use has become an important public health concern worldwide. Its influence is even seen among healthcare students, who rely heavily on digital platforms for academic, professional, and social engagement. Behavioural addiction is linked with excessive social media use and poor psychological well-being [1,2]. Current research suggests that compulsive or maladaptive social media engagement leads to behavioural addiction and feelings of social isolation, which is contradictory to the purpose of these platforms [3,4].

Behavioural addiction to social media is characterised by compulsive use, preoccupation, mood modification, tolerance, withdrawal, conflict, and relapse, which mirror traditional substance addictions [5]. The Bergen Social Media Addiction Scale (BSMAS) is a validated psychometric screening tool used to assess the extent of problematic social media use, focusing on the frequency and psychological impact of social media usage [6]. Healthcare students, who are often under high academic stress and time constraints, may be especially vulnerable to using social media as a coping mechanism, thus increasing their risk for addiction-like behaviours [7].

Social isolation is increasingly linked to the overuse of digital media [8]. The UCLA Loneliness Scale Version 3 is a standardised tool for evaluating subjective feelings of loneliness and social disconnection, providing an important view into how digital interaction may fail to substitute for face-to-face communication [9]. Although young adults are constantly connected, feelings of loneliness, anxiety, and emotional detachment are frequently reported, indicating that online interactions may lead to poor social support systems [10]. This phenomenon of social isolation often affects healthcare students, whose future roles require emotional resilience, empathy, and strong communication skills [11,12].

The mental well-being of healthcare students has direct implications for future healthcare delivery. Prolonged loneliness and psychological stress may lead to poor academic performance, increased susceptibility to burnout, and decreased professional competence [13]. Therefore, exploring the complex interplay between problematic social media use and social isolation in this population is both timely and necessary.

Current literature highlights a bidirectional relationship between these constructs, where excessive use of social media leads to increased feelings of loneliness, which may further contribute to compulsive use. However, research in this domain remains limited among healthcare students, especially in South Asian and developing-country contexts [14]. Given cultural differences in social media usage patterns and family structures, region-specific findings are essential for designing targeted interventions.

This study aligns with Sustainable Development Goal 3: Good Health and Well-Being, as the relationship between social media use-related behavioural addiction and loneliness is an emerging mental health issue. The study will provide evidence for the early identification of at-risk young adult healthcare students and help in the development of targeted mental health support and preventive strategies.

The objectives of the study are clearly defined as follows: (i) Primary Objective: To assess the correlation between problematic social media use and social isolation among healthcare students. (ii) Secondary Objectives: To compare problematic social media use and loneliness among different groups of healthcare students; to identify factors associated with problematic social media use and loneliness among healthcare students; to examine the association of selected sociodemographic variables with problematic social media use and loneliness.

## Materials and methods

Study design and setting

A cross-sectional descriptive study was conducted to explore the influence of social media use on behavioural addiction and social isolation. The study participants comprised undergraduate students admitted to medical and nursing programs at Uttar Pradesh University of Medical Sciences (UPUMS), Saifai, India, between October and November 2024.

Eligibility

A total enumerative sampling technique was used, and all students from the two target programs were invited to participate in the study. The inclusion criteria were undergraduate students enrolled in medical and nursing programs at UPUMS, aged between 18 and 26 years at the time of data collection, with self-reported active use of at least one social media platform, such as Instagram, Facebook, Snapchat, or Telegram, on ≥5 days per week. Participants who provided written consent were included in the study. Students with a self-reported current diagnosis of a psychiatric disorder, such as depression or anxiety, were excluded. Incomplete or illegible questionnaire responses and withdrawal of consent at any stage prior to data analysis were also excluded from the study.

Sample size

The sample size was calculated using the formula $n = \frac{Z^2 P(1-P)}{d^2}$​​​​​​, based on a 17.7% prevalence of social media addiction [15]. The estimated sample size was 224, with a 95% confidence interval and 5% precision. However, only 207 participants agreed to participate in the study, of whom seven submitted responses were excluded due to incomplete data. The final sample size for this study was 200.

Data collection 

All participants who met the inclusion criteria were invited to participate in the study. The purpose of the study was explained, and its potential benefits to healthcare education were discussed. Further, confidentiality, the right to withdraw, and anonymity of responses were explained. Participants were recruited after their class times. Those who provided written consent were given paper-based questionnaires. They were allowed 45 minutes to complete the questionnaire. The completed questionnaires were filled out by the students and collected in person by a member of the research team. Data were collected only in the presence of research members. 

Study tools

Data were collected using a self-structured demographic tool that included sociodemographic variables such as age, gender, undergraduate program, living arrangement, family relationship detachment, type of family, most used apps/websites, most used gadgets, duration of social media use per day, and reasons for social media use (see Appendix 1). Family relationship detachment was assessed using a single-item question with response options of “Yes” or “No.” Here, family detachment refers to an individual’s perception of emotional distance or lack of connectedness with family. The response was recorded as yes or no for ease of administration, and no further categorisation was done.

In addition, behavioural addiction related to social media was assessed using the BSMAS, a validated screening tool used to measure problematic social media use based on the six core components of behavioural addiction: salience, mood modification, tolerance, withdrawal, conflict, and relapse. It consists of six items, each rated on a five-point Likert scale ranging from 1 (very rarely) to 5 (very often), evaluating participants’ behaviour over the past 12 months. A higher total score indicates greater dependence on social media. The reliability is reported with Cronbach’s alpha = 0.88.

Social isolation was assessed using the UCLA Loneliness Scale Version 3. The UCLA Loneliness Scale (Version 3) is a 20-item self-report scale designed to measure subjective feelings of loneliness and social isolation. Participants rated each item as O (“I often feel this way”), S (“I sometimes feel this way”), R (“I rarely feel this way”), or N (“I never feel this way”). The reliability of this standardised tool is 0.89. The total score ranges from 20 to 80. Scoring is categorised as follows: 20-34 (low loneliness), 35-49 (moderate loneliness), 50-64 (moderately high loneliness), and 65-80 (high loneliness) [6,9].

Statistical analysis

Data analysis was done using IBM SPSS Statistics for Windows, Version 26 (Released 2018; IBM Corp., Armonk, NY, USA), incorporating descriptive and inferential statistics. Descriptive statistics such as frequency, percentage, mean, and standard deviation were used to summarise the study variables. The Mann-Whitney U test was used to compare social media addiction and loneliness scores among undergraduate students because the data did not follow a normal distribution, as assessed by the Shapiro-Wilk test. Spearman’s rank correlation analysis was utilised to examine the relationship between problematic social media use and loneliness. Multiple linear regression was used to examine the extent to which demographic characteristics explain the variation in BSMAS and loneliness scores. Statistical significance was set at p < 0.05. In this study, regression assumptions included linearity, normality of residuals, homoscedasticity, and multicollinearity. Multicollinearity was checked using the variance inflation factor (VIF). Further detailed diagnostic output was not included. 

Ethical considerations

The ethical approval was obtained from the Institutional Ethics Committee of UPUMS, Saifai (IEC No. 164/2023-24), and adhered to the principles of the Declaration of Helsinki. Voluntary participation and informed consent were strictly followed. All filled records were stored securely, and consent was separated from the questionnaire data to maintain confidentiality. Participants’ personal information was not obtained throughout the study; coding of all responses was done, and the informed consent was separated from the data.

## Results

The study included 200 participants. Among them, 117 (58.5%) were in the age category of 21-23 years, and 102 (51%) were females. The participants were equally distributed between MBBS and Nursing students. A large majority of students, 193 (96.5%), were residing in hostels, although 172 (86%) of them reported no family detachment. Most students, 151 (75.5%), belonged to nuclear families. According to participants, Instagram was identified as the most frequently used social media platform by 100 (50%) of students. The majority of participants, 194 (97%), reported smartphones as their primary gadgets for social media use. The duration of social media use was more than three hours per day for 76 (38%) participants. Entertainment was the most common reason for social media use among 111 (55.5%) participants (Table 1).

**Table 1:** Demographic profile (n = 200)

S. No.	Demographic Profile	n (%)
1.	Age (in years)	
a.	18-20	57 (28.5%)
b.	21-23	117 (58.5%)
c.	24-26	26 (13%)
2.	Gender	
a.	Female	102 (51%)
b.	Male	98 (49%)
3.	Undergraduate program	
a.	MBBS	100 (50%)
b.	Nursing	100 (50%)
4.	Living set up	
a.	Hostel	193 (96.5%)
b.	Family members	7 (3.5%)
5.	Family relationship detachment	
a.	No	172 (86%)
b.	Yes	28 (14%)
6.	Type of family	
a.	Joint	49 (24.5%)
b.	Nuclear	151 (75.5%)
7.	Most used apps/websites	
a.	Facebook	5 (2.5%)
b.	WhatsApp	77 (38.5%)
c.	Instagram	100 (50%)
d.	Snapchat	3 (1.5%)
e.	You tube	15 (7.5%)
8.	Most used gadgets	
a.	Laptop	4 (2%)
b.	Tablet	2 (1%)
c.	Smart phone	194 (97%)
9.	Duration of social media use/day	
a.	<1 hour	20 (10%)
b.	1-2 hours	42 (21%)
c.	2-3 hours	62 (31%)
d.	>3 hours	76 (38%)
10.	Reason for social media use	
a.	Communication	29 (14.5%)
b.	Entertainment	111 (55.5%)
c.	Self-expression	2 (1%)
d.	Education content	29 (14.5%)
e.	Fear of missing out	5 (2.5%)
f.	Information	24 (12%)

Comparison of problematic social media use and loneliness using the Mann-Whitney U test

BSMAS Score

Nursing students showed a score of 16.1 ± 4.36, slightly higher than MBBS students (15.5 ± 5.05). The Mann-Whitney U test showed a U statistic of 4660 with a p-value of 0.405, indicating that the difference in social media addiction scores between the two groups was not statistically significant. The effect size (r = 0.068) indicates a very small value, pointing to a minimal practical difference between the groups.

UCLA Loneliness Score

The mean loneliness score was higher among Nursing students (45 ± 13.37) in comparison to MBBS students (41 ± 14.21). The Mann-Whitney U test showed a U statistic of 4138 with a p-value of 0.035, indicating that the difference in loneliness scores was statistically significant. The effect size (r = 0.173) shows a difference, with Nursing students experiencing slightly higher levels of loneliness compared to MBBS students (Table 2).

**Table 2:** Comparison of problematic social media use and loneliness using the Mann-Whitney U test *p < 0.05 indicates statistical significance.

Variables	MBBS Students (Mean ± SD)	Nursing Students (Mean ± SD)	U Statistic	p-value	Effect Size (r)
BSMAS Score	15.5 ± 5.05	16.1 ± 4.36	4660	0.405	0.068
Loneliness	41 ± 14.21	45 ± 13.37	4138	0.035*	0.173

Correlation between problematic social media use and loneliness

Correlation between BSMAS and UCLA loneliness scores was assessed using Spearman’s correlation analysis, which showed a small-to-moderate but significant positive correlation between problematic social media use and loneliness, rs(198) = 0.325, p < 0.001, indicating that higher social media usage was associated with greater loneliness (Table 3).

**Table 3:** Correlation between problematic social media use and loneliness *p < 0.05 indicates statistical significance.

Variables	Spearman’s Rho (Þ)	df	p-value
Social Media Addiction - Loneliness	0.33	198	<0.001*

Determinants of problematic social media use and loneliness among undergraduate students

To determine the extent to which demographic characteristics explain the variation in BSMAS and loneliness scores, multiple linear regression was used. For problematic social media use (BSMAS score), duration of daily social media use was a statistically significant predictor (β = 0.316, t = 4.696, p < 0.001). The overall model showed the proportion of variance explained (R² = 0.298; adjusted R² = 0.118), indicating limited explanatory power for the model. The positive coefficient shows that participants who spend more time on social media have significantly higher BSMAS scores, whereas age (p = 0.135) and other demographic variables were not statistically significant predictors.

For loneliness scores, the regression analysis showed that family relationship detachment was a significant predictor (β = 0.21, t = 2.98, p = 0.003). Participants reporting detachment from family members showed higher loneliness scores compared to those who did not report family detachment. The model explained a modest proportion of variance (adjusted R² = 0.195), whereas duration of social media use (p = 0.097) and age (p = 0.429) showed positive but non-significant associations (Table 4).

**Table 4:** Determinants of problematic social media use and loneliness among undergraduate students Note: R = multiple correlation coefficient; R² = coefficient of determination; SE = standard error; B = unstandardised coefficient; β = standardised coefficient; * statistically significant where p < 0.05 (two-tailed).

Variables	Predictor	B	SE	t	p	β	R	R^2^	Adjusted R^2^
BSMAS Score	Duration of social media use/day	1.57	0.33	4.69	<0.001*	0.31	0.546	0.298	0.118
	Age (years)	0.80	0.53	1.50	0.135	0.10			
Loneliness Score	Family relationship detachment	8.89	2.98	2.98	0.003*	0.21	0.460	0.211	0.195
	Duration of social media use/day	1.74	1.05	1.66	0.097	0.11			
	Age (years)	1.32	1.67	0.79	0.429	0.05			

## Discussion

The present study assesses the influence of social media use on behavioural addiction and social isolation tendencies among healthcare students. Findings showed that Instagram is the most frequently used social media platform, followed by WhatsApp. Similar findings are seen in surveys conducted in India, which report 516.92 million active Instagram users, with WhatsApp being the most used messaging site. Previous studies likewise highlight Instagram and YouTube as the most used social networks among healthcare students, often used as learning tools [16]. The smartphone emerges as the most frequently used gadget among participants; prior research also identifies moderate levels of smartphone addiction among young adults [17,18].

Consistent with previous research among adolescents, it is suggested that spending more than three hours per day on social media increases the risk of mental health issues, particularly internalising problems [19]. Similarly, participants in this study also revealed that the social media threshold was exceeded in their daily use. However, contrary to previous research focusing on social or academic motives, the main reason for social media engagement among participants in this study was entertainment. The finding aligns with the work of Bauer and Tian (2024), which showed that entertainment, information seeking, and other antecedent variables have a significant influence on psychological states and behavioural engagement with social media [20,21].

A small effect size (r = 0.068) was observed, indicating a minimal practical difference in BSMAS scores between MBBS and B.Sc. Nursing students. Comparable global studies have reported similar findings, such as a study conducted among 340 nurses at Cairo University, which revealed that 60.59% of participants were moderately addicted to social media, along with a significant association between social media use and age [22]. Studies in Dubai reported that the majority of medical students had moderate to severe levels of social media addiction, while a study conducted in Korea revealed a negative correlation between psychological well-being and social media addiction [23,24].

Furthermore, Norwegian studies have highlighted that social media addiction among adolescents is strongly associated with the manifestation of anxiety and depression [25]. Excessive engagement with social media platforms has also been linked to poor academic outcomes, including lower grades and reduced study time [26].

The present study also found that a higher UCLA loneliness score is associated with nursing students, consistent with a network analysis approach study, which also showed a higher prevalence of loneliness among nursing students [27]. A strong correlation was reported between social media addiction and loneliness, aligning with prior findings showing a positive correlation between smartphone addiction and loneliness [28-31].

Regression analyses showed that the duration of daily social media use was the strongest predictor of social media addiction, corroborating earlier reports that excessive use of social media increases compulsive usage tendencies. Whereas demographic variables such as age and gender did not significantly contribute, behavioural factors primarily influenced addictive tendencies [32-34]. Conversely, other studies have reported that demographic characteristics such as female gender, younger age, and academic performance impact social media addiction [35,36].

For loneliness, family relationship detachment emerged as a significant predictor, explaining approximately 21% of the variance. Students reporting weaker familial bonds exhibited substantially higher loneliness scores, supporting previous findings that strong family connectedness leads to decreased social isolation and emotional distress [37].

Although the duration of social media use showed a positive trend, it was not a significant predictor of loneliness. This suggests that while the duration of social media use contributes to loneliness, familial ties play a more decisive role in the development of emotional well-being.

Limitations

The study has several limitations, such as a cross-sectional design, which limits the ability to draw causal conclusions from the data. Participants’ data may have introduced self-reported bias. The study was conducted in a single setting and specific academic context, which further restricts generalisability. Family relationship detachment was assessed through a self-structured questionnaire. The majority of participants were residing in hostels, resulting in low variability in living context and potentially inflating setting-specific effects. Although a diagnosed psychiatric illness was excluded, the possibility of undiagnosed or subclinical psychological conditions cannot be ruled out. The model’s R² value indicates that several influencing factors remain unexplored. These limitations may hinder the interpretation of the results, especially in terms of the generalisability of the findings.

## Conclusions

Greater time spent on social media is significantly associated with problematic social media use, while weaker family relationships are associated with higher loneliness. There is a need for targeted interventions such as digital literacy, promotion of balanced social media habits, and supportive interpersonal relationships to improve emotional well-being. Further research should focus on analysing the underlying behavioural and emotional factors contributing to problematic social media use. Future studies can include validated tools such as PHQ-9, GAD-7, and PSS, along with longitudinal designs, in order to develop focused programs to promote healthy and balanced social media use.

## Appendices

Appendix 1

**Table 5:** Demographic profile Instructions: Tick (✓) only one option for each statement.

S. No.	Demographic Profile	n (%)
			1.	Age (in years)	
a.	18-20	( )
b.	21-23	( )
c.	24-26	( )
2.	Gender	
a.	Female	( )
b.	Male	( )
3.	Undergraduate program	
a.	MBBS	( )
b.	Nursing	( )
4.	Living set up	
a.	Hostel	( )
b.	Family members	( )
5.	Family relationship detachment	
a.	No	( )
b.	Yes	( )
6.	Type of Family	
a.	Joint	( )
b.	Nuclear	( )
7.	Most used apps/websites	
a.	Facebook	( )
b.	WhatsApp	( )
c.	Instagram	( )
d.	Snapchat	( )
e.	You tube	( )
8.	Most used Gadgets	
a.	Laptop	( )
b.	Tablet	( )
c.	Smart phone	( )
9.	Duration of social media use/day	
a.	<1 hour	( )
b.	1-2 hours	( )
c.	2-3 hours	( )
d.	>3 hours	( )
10.	Reason for social media use	
a.	Communication	( )
b.	Entertainment	( )
c.	Self-expression	( )
d.	Education content	( )
e.	Fear of missing out	( )
f.	Information	( )
